# Prediction of pre‐eclampsia‐related complications in women with suspected or confirmed pre‐eclampsia: development and internal validation of clinical prediction model

**DOI:** 10.1002/uog.23142

**Published:** 2021-10-06

**Authors:** L. Saleh, M. M. Alblas, D. Nieboer, R. I. Neuman, Y. Vergouwe, I. A. Brussé, J. J. Duvekot, E. W. Steyerberg, H. J. Versendaal, A. H. J. Danser, A. H. van den Meiracker, K. Verdonk, W. Visser

**Affiliations:** ^1^ Department of Internal Medicine Division of Vascular Medicine and Pharmacology, Erasmus MC Rotterdam The Netherlands; ^2^ Department of Obstetrics and Gynecology Division of Obstetrics and Prenatal Medicine, Erasmus MC Rotterdam The Netherlands; ^3^ Centre for Medical Decision Sciences, Department of Public Health Erasmus MC Rotterdam The Netherlands; ^4^ Department of Obstetrics and Gynecology Division of Obstetrics and Prenatal Medicine, Maasstad Ziekenhuis Rotterdam The Netherlands

**Keywords:** PlGF, prediction model, pre‐eclampsia, sFlt‐1

## Abstract

**Objective:**

A model that can predict reliably the risk of pre‐eclampsia (PE)‐related pregnancy complications does not exist. The aim of this study was to develop and validate internally a clinical prediction model to predict the risk of a composite outcome of PE‐related maternal and fetal complications within 7, 14 and 30 days of testing in women with suspected or confirmed PE.

**Methods:**

The data for this study were derived from a prospective, multicenter, observational cohort study on women with a singleton pregnancy and suspected or confirmed PE at 20 to < 37 weeks' gestation. For the development of the prediction model, the possible contribution of clinical and standard laboratory variables, as well as the biomarkers soluble fms‐like tyrosine kinase‐1 (sFlt‐1), placental growth factor (PlGF) and their ratio, in the prediction of a composite outcome of PE‐related complications, consisting of maternal and fetal adverse events within 7, 14 and 30 days, was explored using multivariable competing‐risks regression analysis. The discriminative ability of the model was assessed using the concordance (c‐) statistic. A bootstrap validation procedure with 500 replications was used to correct the estimate of the prediction model performance for optimism and to compute a shrinkage factor for the regression coefficients to correct for overfitting.

**Results:**

Among 384 women with suspected or confirmed PE, 96 (25%) had an adverse PE‐related outcome at any time after hospital admission. Important predictors of adverse PE‐related outcome included sFlt‐1/PlGF ratio, gestational age at the time of biomarker measurement and protein‐to‐creatinine ratio as continuous variables. The c‐statistics (corrected for optimism) for developing a PE‐related complication within 7, 14 and 30 days were 0.89, 0.88 and 0.87, respectively. There was limited overfitting, as indicated by a shrinkage factor of 0.91.

**Conclusions:**

We propose a simple clinical prediction model with good discriminative performance to predict PE‐related complications. Determination of its usefulness in clinical practice awaits further investigation and external validation. © 2020 The Authors. Ultrasound in Obstetrics & Gynecology published by John Wiley & Sons Ltd on behalf of International Society of Ultrasound in Obstetrics and Gynecology.

**CONTRIBUTION**

**What are the novel findings of this work?**

We present a prediction model to calculate simply the absolute risk of developing a composite outcome of pre‐eclampsia (PE)‐related pregnancy complications, consisting of both maternal and fetal adverse events, in the subsequent 7, 14 and 30 days, in women with suspected or confirmed PE. Our model is not limited to diagnosing PE but also addresses fetal risk.

**What are the clinical implications of this work?**

Our model could help physicians to identify low‐risk patients with suspected or confirmed PE who could be offered expectant management, leading to a decrease in the number and duration of admissions to the obstetric ward and potentially a reduction in costs, while simultaneously not compromising maternal and fetal health outcomes.

## INTRODUCTION

Pre‐eclampsia (PE), a pregnancy‐specific syndrome traditionally characterized by elevated blood pressure and proteinuria, affects approximately 5% of all pregnancies and is an important cause of death and complications for women and their babies^1^. The current management of patients with suspected or confirmed PE requires resource‐intensive measures such as hospital admission for close monitoring of maternal blood pressure and laboratory trends due to the risk of eclampsia, liver, lung and kidney damage, intrauterine growth restriction, prematurity and maternal and fetal demise^2^, ^3^. Well‐known risk factors for PE include a history of PE, nulliparity, advanced maternal age and pre‐existing conditions such as hypertension, pregestational diabetes, obesity and chronic kidney disease, but the precise cause of PE remains unclear. Ample evidence suggests a role for placental overproduction of soluble fms‐like tyrosine kinase‐1 (sFlt‐1), which results in an antiangiogenic state due to the inhibition of vascular endothelial growth factors, including placental growth factor (PlGF)^4^. Several models have been developed to calculate the predicted risk of PE, some of which include sFlt‐1 and PlGF as predictors^5^, ^6^, but a prediction model that could estimate the risk of pregnancy complications rather than diagnosing the heterogeneous PE syndrome does not currently exist. We have reported previously that incorporation of sFlt‐1, PlGF and the sFlt‐1/PlGF ratio into a model based on clinical and traditional laboratory variables has substantial value in the prediction of both maternal and fetal complications as well as pregnancy prolongation in patients with suspected or confirmed PE^7^. We have also demonstrated that incorporation of sFlt‐1 and PlGF values separately is associated with better prediction of complications as compared with proposed sFlt‐1/PlGF ratio cut‐offs^7^. As a follow‐up of these findings, our aim was to develop and validate internally a clinical model to predict the risk of developing a composite outcome of PE‐related pregnancy complications within 7, 14 and 30 days in women with suspected or confirmed PE.

## METHODS

### Study design

Women with a singleton pregnancy from varied ethnic backgrounds were recruited into a prospective multicenter cohort study (PRE‐RATIO study^7^) at three Dutch hospitals (Erasmus MC and Maasstad Hospital in Rotterdam, Reinier de Graaf Hospital in Delft). The same study protocol and data collection forms were used at each center. All women provided written informed consent to participate in the study, which was approved by the local research ethics committee (MEC‐2013‐202).

### Study population

The inclusion criteria for the original cohort were women aged ≥ 18 years with a singleton pregnancy at a gestational age of ≥ 20 weeks who had PE, new onset of elevated blood pressure, aggravation of pre‐existing hypertension, new onset of proteinuria, aggravation of pre‐existing proteinuria, decreased platelet levels, increased liver enzymes or one or more other reason(s) for clinical suspicion of PE such as epigastric pain, severe headache or excessive edema/severe swelling (of the face or extremities). PE was defined according to the Dutch guideline of De Nederlandse Vereniging voor Obstetrie en Gynaecologie (NVOG) as new onset of hypertension (systolic blood pressure (SBP) of ≥ 140 mmHg and/or diastolic blood pressure (DBP) of ≥ 90 mmHg) and new onset of proteinuria (protein‐to‐creatinine ratio (PCR) ≥ 30 mg/mmol, 24‐h urine ≥ 0.3 g/day or dipstick protein ≥ 2+) at or after 20 weeks' gestation^8^. At the Erasmus MC and Maasstad Hospital, PCR was initially determined and, when increased (≥ 30 mg/mmol), 24‐h urine was then collected. In Reinier de Graaf Hospital, PCR was determined after a positive dipstick test (2+).

HELLP syndrome was defined as hemolysis (haptoglobin < 0.1 g/L), elevated liver enzymes (alanine transaminase > 41 U/L or lactate dehydrogenase (LDH) > 321 U/L) and low platelet count (< 100 × 10^9^/L), with or without PE. Partial HELLP syndrome was defined as the presence of two of the HELLP criteria. Superimposed PE was diagnosed in women with chronic hypertension who had new onset of proteinuria, a sudden increase in blood pressure, appearance of thrombocytopenia and increased liver enzymes, or a sudden increase in proteinuria in patients with pre‐existing proteinuria. Isolated new‐onset hypertension at or after 20 weeks was defined as gestational hypertension (GH). Patients with suspicion of PE but without GH were defined as having no hypertensive disease of pregnancy^8^. Eligible patients for this subanalysis were women with suspected or confirmed PE at a gestational age of 20 to < 37 weeks. Women with (partial) HELLP syndrome or fetal death at study entry were excluded.

### Outcome measures

A composite outcome of PE‐related pregnancy complications, consisting of maternal and fetal adverse events, was established and defined as occurrence of at least one maternal or fetal complication. Maternal complications were defined as: acute renal failure (absolute increase in serum creatinine concentration of ≥ 0.3 mg/dL (26.5 µmol/L) from baseline, a ≥ 50% increase in serum creatinine or oliguria with < 0.5 mL/kg/h over a period of 6 h), cerebral hemorrhage/edema or infarction, death, eclampsia, development of (partial) HELLP syndrome, pulmonary edema, placental abruption, visual disturbances and subcapsular liver hematoma. Fetal complications were defined as fetal death or fetal distress requiring immediate delivery. The duration until occurrence of a complication was defined as the number of days from study inclusion and blood sampling until occurrence of the complication. The occurrence of the composite outcome within the subsequent 7, 14 and 30 days was assessed. As agreed with the obstetricians of the project group, patients with a score of < 5% were considered to have a very low risk of developing a complication within the forthcoming 7 days and therefore would not require hospitalization and could be followed at the outpatient clinic. Patients with a risk of 5% or higher would be admitted to hospital or remain in hospital.

The treating obstetricians filled in forms in which they had to answer the following questions, taking into account data available on traditional variables: ‘What is the chance of developing PE within this pregnancy? (0–100%)’; ‘What is the severity of the disease? (0–10)’; and ‘For how long can this pregnancy be temporized? (0, 1, 2–7, 8–14, > 14 days)’. Clinical findings, physical examination, laboratory test results, maternal and fetal complications (diagnosed by the treating physicians) and background data of the patients were obtained from patient's electronic medical records.

### Serum samples

For the measurement of sFlt‐1 and PlGF, serum was prepared from venous whole blood, and assays were performed at the clinical laboratory of the Erasmus MC using an automated biochemistry analyzer (Cobas 6000, e‐module; Roche Diagnostics, Mannheim, Germany). Samples were stored at –80°C until analysis after completion of the study.

### Statistical analysis

Patient characteristics are given as median (interquartile range (IQR)) for continuous variables and as *n* (%) for categorical variables. Missing data on candidate predictors were imputed using multivariate imputation by chained equations. Results from statistical analyses were pooled using Rubin's rules^9^. Non‐linearity of associations between continuous candidate predictors and the risk of developing complications was assessed using logarithmic transformations.

A Fine–Gray semiparametric proportional hazards model was used to evaluate the effect of predictors on time to develop complications, adjusting for the competing risk of delivery. The candidate predictors considered were sFlt‐1, PlGF, sFlt‐1/PlGF ratio, gestational age, proteinuria (PCR ≥ 30 mg/mmol or ≥ 300 mg/24 h or 2+ dipstick), DBP (SBP was not considered because of its strong correlation with DBP, and a model with SBP rather than DBP gave a similar fit), LDH, uric acid and platelet count. The relationship between each predictor variable and the composite outcome was assessed using univariable logistic regression analysis. The effects were quantified using subdistribution hazard ratios. Subsequently, we developed a model to estimate the absolute risk of developing a pregnancy complication (i.e. the composite outcome of PE‐related pregnancy complications) using the cumulative incidence function.

To ensure an accurate prediction model, candidate predictors were evaluated when at least 10 complications had been registered. Subsequently, backward selection was applied to limit the number of predictors in the final prediction model. Model specification was based on the Akaike Information Criterion in a backward selection procedure. This is equivalent to exclusion of candidate predictors with *P* > 0.157. The discriminative ability of the developed model was assessed using the concordance (c)‐statistic. Discrimination refers to how well the model distinguishes between those with and those without PE‐related complications at specific timepoints. The c‐statistic ranges from 0.5 for a model equivalent to a coin toss to 1.0 for a model with perfect discrimination.

The developed model was validated internally using bootstrap resampling. Prediction models were developed in bootstrap samples, with selection and coefficient estimation repeated in each. Such bootstrap validation maximizes statistical efficiency and directly validates the final model. A shrinkage factor was obtained for correction of calibration of predictions, and an optimism‐corrected c‐statistic was calculated. A prediction model was constructed in Excel (Microsoft Corp., Redmond, WA, USA) to allow the final model to be implemented easily in clinical practice. We used SPSS Statistics 21 (IBM Corp., Armonk, NY, USA) and R Software version 3.3.2 (R foundation for statistical computing, Vienna, Austria; cmprsk and riskRegression libraries) for statistical analysis.

## RESULTS

Of the 620 women with suspected or confirmed PE from the original cohort, 422 had a gestational age between 20 and < 37 weeks and were eligible for inclusion in the study, of whom 38 were excluded owing to the presence of (partial) HELLP syndrome or fetal death at study entry (Figure S1). We therefore included 384 singleton pregnancies with suspected or confirmed PE at 20 to < 37 weeks' gestation, of which 220 (57%) had a gestational age below 34 weeks (Table 1). Among these, 68 (18%) women had GH, 130 (34%) had PE or superimposed PE and 186 (48%) had no hypertensive disease of pregnancy at study entry (Table 1). The median time from inclusion to delivery was 21 days (IQR, 8–41 days). In total, 96 (25%) pregnancies developed composite adverse PE‐related maternal/fetal outcome at any time after hospital admission (Table 2). The percentage of missing values was low (1–3%), except in the case of the PCR, for which it was 34%. In patients without a PCR measurement, either 24‐h urine protein or the dipstick test was assessed, and this was taken into account in the statistical imputation procedure.

**Table 1:** Characteristics of 384 pregnancies with suspected or confirmed pre‐eclampsia, from the PRE‐RATIO study^7^

Characteristic	Value
Age (years)	31 (27–36)
Gestational age at enrolment < 34 weeks	220 (57.3)
Nulliparous	200 (52.1)
Current smoker	39 (10.2)
Ethnicity	
White	246 (64.1)
Black	73 (19.0)
Other	65 (16.9)
History of pre‐eclampsia	74 (19.3)
Pre‐existing hypertension	101 (26.3)
Pre‐existing proteinuria	23 (6.0)
Clinical findings at time of admission	
Systolic blood pressure (mmHg)	138 (126–150)
Diastolic blood pressure (mmHg)	86 (80–95)
Protein‐to‐creatinine ratio (mg/mmol)	30 (14–82)
Lactate dehydrogenase (U/L)	184 (160–217)
Alanine transaminase (U/L)	15 (11–23)
Creatinine (µmol/L)	56 (49–65)
Uric acid (mmol/L)	0.28 (0.23–0.34)
Platelet count (10^9^/L)	233 (186–279)
sFlt‐1 (pg/mL)	3137 (1721–6843)
PlGF (pg/mL)	139 (62–354)
sFlt‐1/PlGF ratio	23 (5–90)
Diagnosis at inclusion	
No hypertensive disease of pregnancy	186 (48.4)
Gestational hypertension	68 (17.7)
Pre‐eclampsia	86 (22.4)
Superimposed pre‐eclampsia	44 (11.5)

Data are given as median (interquartile range) or *n* (%).

PlGF, placental growth factor; sFlt‐1, soluble fms‐like tyrosine kinase‐1.

**Table 2:** Outcome and maternal and fetal complications in 384 pregnancies with suspected or confirmed pre‐eclampsia

Outcome	Value
Gestational age at birth (weeks)	37.1 (34.2–38.2)
Preterm delivery < 34 weeks	85 (22.1)
Preterm delivery at 34–37 weeks	73 (19.0)
Female neonate	186 (48.4)
Birth weight (g)	2813 (1965–3329)
Enrolment‐to‐delivery interval (days)	21 (8–41)
Duration of hospitalization (days)	6 (3–13)
Final diagnosis	
No hypertensive disease of pregnancy	143 (37.2)
Gestational hypertension	64 (16.7)
Pre‐eclampsia	102 (26.6)
Superimposed pre‐eclampsia	53 (13.8)
(Partial) HELLP syndrome	22 (5.7)
Maternal complications*	
(Partial) HELLP syndrome	22 (5.7)
Placental abruption	1 (0.3)
Pulmonary edema	7 (1.8)
Renal insufficiency	2 (0.5)
Visual disturbance	3 (0.8)
Fetal complications	
Fetal distress requiring elective CS	55 (14.3)
Fetal death	9 (2.3)
Composite adverse maternal/fetal outcome†	96 (25.0)

Data are given as median (interquartile range) or *n* (%).

* Cerebral hemorrhage/edema or infarction, maternal death, eclampsia and subcapsular liver hematoma are not listed in the table because these pre‐eclampsia‐related complications did not occur in the study population.

† Defined as at least one maternal or fetal complication.

CS, Cesarean section.

Transformation with the natural logarithm provided the best fit for the association of PlGF, sFlt‐1/PlGF ratio, proteinuria, PCR, DBP and platelet count with the composite outcome, while a linear relationship of sFlt‐1, gestational age, LDH and uric acid with the composite outcome was a reasonable approximation. The final multivariable model included sFlt‐1/PlGF ratio, gestational age at the time of biomarker measurement and PCR as continuous variables. The sFlt‐1/PlGF ratio and gestational age at the time of biomarker measurement were the strongest predictors for PE‐related complications (*P* < 0.001 for both) (Table 3).

**Table 3:** Subdistribution hazard ratios of the multivariable model for prediction of composite adverse pre‐eclampsia‐related maternal/fetal outcome in women with suspected or confirmed pre‐eclampsia

Variable	Hazard ratio (95% CI)	*P*
GA at biomarker measurement (in weeks)	0.99 (0.98–0.99)	< 0.001
^2^log PCR (in mg/mmol)*	1.15 (1.01–1.32)	0.03
^2^log sFlt‐1/PlGF ratio*	1.49 (1.39–1.63)	< 0.001

* Variables were transformed with ^2^log such that hazard ratios refer to a doubling of variables.

GA, gestational age; PCR, protein‐to‐creatinine ratio; sFlt‐1/PlGF, soluble fms‐like tyrosine kinase‐1/placental growth factor ratio.

As illustrated in Figure S2, a patient with suspected PE at a gestational age of 35 + 1 weeks with a PCR of 10 mg/mmol and a sFlt‐1/PlGF ratio of 10 has a predicted probability of 2.6% for a complication within 7 days. This risk increases to 4.0% and 4.9% in the subsequent 7 and 23 days, respectively (Figure S2b). If the same patient had an increased PCR of 38 mg/mmol, the predicted probability for a complication within 7 days would increase to 3.3% (Figure S2c), while a solely elevated sFlt‐1/PlGF ratio of 38 in the same patient increases the risk to 5.2% (Figure S2d).

The c‐statistic (corrected for optimism) of the model for prediction of developing a PE‐related complication within 7 days was 0.89, meaning that the model has an excellent ability to discriminate between patients who will develop a PE‐related complication and those who will not. This was similarly found for predicting the development of a PE‐related complication within 14 and 30 days, with c‐statistics (corrected for optimism) of 0.88 and 0.87, respectively. The model was validated internally by performing a bootstrap validation procedure with 500 replications in which we obtained a shrinkage factor of 0.91, meaning there was limited overfitting. The regression coefficient for future patients should be multiplied by 0.91.

Only one out of the 170 (0.6%) pregnancies in the low‐risk group (< 5% risk) developed an adverse PE‐related outcome within 7 days after entry to the study (Table 4). This 29‐year‐old primigravida was included in the study at 34 + 6 weeks' gestation and delivered 3 days later by emergency Cesarean section because of suspicion of fetal distress due to suboptimal cardiotocography. Patients in the high‐risk group delivered earlier, had lower birth‐weight centile and developed significantly more often composite adverse PE‐related maternal/fetal outcomes than did those in the low‐risk group.

**Table 4:** Characteristics and occurrence of adverse maternal and fetal outcomes in 384 pregnancies with suspected or confirmed pre‐eclampsia, according to whether they were low (< 5%) or high (≥ 5%) risk based on the prediction model

Variable	Low risk (*n* = 170)	High risk (*n* = 214)
Maternal age (years)	32 (27–35)	31 (27–36)
Nulliparous	76 (44.7)	124 (57.9)
GA at enrolment (weeks)	34.0 (30.2–35.3)	31.2 (27.0–35.0)*
GA at enrolment < 34 weeks	81 (47.6)	140 (65.4)*
GA at birth (weeks)	38.1 (37.0–39.0)	35.2 (30.2–37.0)*
Preterm delivery < 34 weeks	4 (2.4)	81 (37.9)*
Preterm delivery at 34–37 weeks	17 (10.0)	56 (26.2)*
Female neonate	81 (47.6)	105 (49.1)
Birth weight < 10^th^ percentile	12 (7.1)	51 (23.8)*
Enrolment‐to‐delivery interval (days)	18 (8–39)	13 (4–26)*
Maternal complication within 7 days†		
(Partial) HELLP syndrome	0 (0)	7 (3.3)
Placental abruption	0 (0)	1 (0.5)
Pulmonary edema	0 (0)	3 (1.4)
Renal insufficiency	0 (0)	1 (0.5)
Visual disturbance	0 (0)	1 (0.5)
Fetal complications within 7 days after enrolment		
Fetal distress requiring elective CS	1 (0.6)	53 (24.8)*
Fetal death	0 (0)	9 (4.2)*
Composite adverse maternal/fetal outcome within 7 days after enrolment	1 (0.6)	53 (24.8)*

Data are given as median (interquartile range) or *n* (%).

* *P* < 0.05 compared with low‐risk group.

† Cerebral hemorrhage/edema or infarction, maternal death, eclampsia and subcapsular liver hematoma are not listed in the table because these pre‐eclampsia‐related complications did not occur in the study population.

CS, Cesarean section; GA, gestational age.

**Figure 1:**
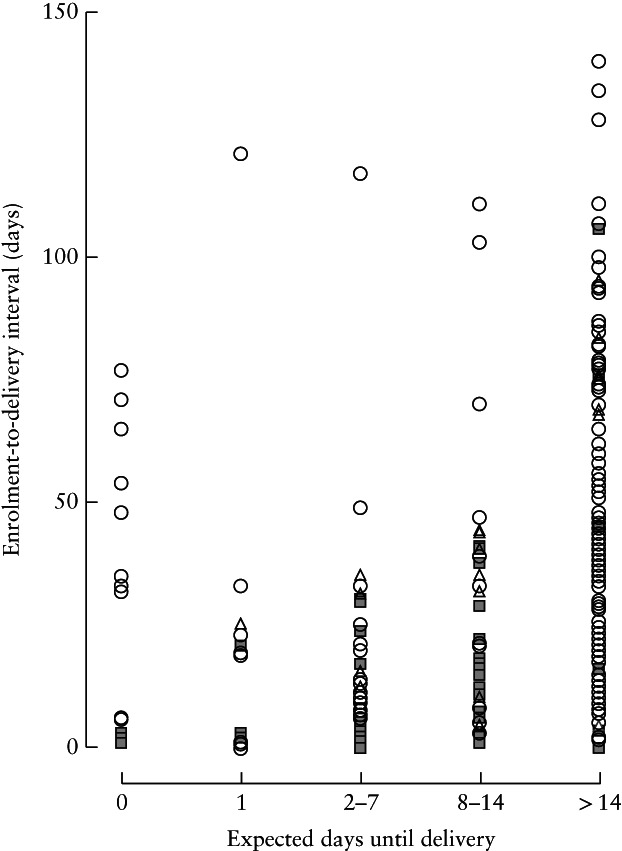
Expected number of days after inclusion until delivery, as predicted by physician, *vs* observed number of days from study entry until delivery, in 323 women with suspected or confirmed pre‐eclampsia, according to diagnosis at study entry: gestational hypertension (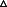), no hypertensive disease of pregnancy (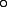) or (superimposed) pre‐eclampsia (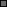).

The predictions of the treating obstetricians for how long the pregnancy would continue were categorized as 0, 1, 2–7, 8–14 and > 14 days (Figure 1, Table 5). The results show that obstetricians predicted poorly the number of days until delivery. None of the obstetricians correctly predicted patients delivering immediately (0 days); the physicians thought that these patients would give birth at least one day later in all cases. In the group of patients who gave birth at 2–7 days, 8–14 days and > 14 days after study entry, obstetricians were correct in, respectively, 35% (18/51), 16% (10/61) and 69% (134/195) of cases.

**Table 5:** Expected number of days after inclusion until delivery, as predicted by obstetrician, *vs* observed number of days from study entry until delivery, in 323 women with suspected or confirmed pre‐eclampsia

Observed days until delivery	Expected days until delivery				
	0	1	2–7	8–14	> 14
0	0	4	1	0	1
1	2	3	4	1	—
2–7	4	6	18	13	10
8–14	0	0	21	10	30
> 14	9	6	20	26	134

Data for 323/384 women are presented owing to missing forms.

## DISCUSSION

We present the development and internal validation of a clinical model for predicting a composite outcome of PE‐related complications, consisting of maternal and fetal adverse events, within 7, 14 and 30 days, in women with suspected or confirmed PE. Continuous values of sFlt‐1/PlGF ratio, PCR and gestational age at the time of blood sampling for biomarker measurements were strong predictors of the development of composite adverse PE‐related outcome. Internal validation, using bootstrap analysis, showed that the model provides excellent discrimination between pregnancies with a high or low risk of developing a complication. The resulting risk predictions for a complication within 7, 14 and 30 days after biomarker measurements could aid in key clinical decisions that are not addressed by existing single‐time‐point prognostic criteria.

The final model, consisting of gestational age, PCR and sFlt‐1/PlGF ratio, for the prediction of a PE‐related complication within 7 days, yielded a higher c‐statistic than that achieved by other models investigated previously in this population. For clinical practice, we translated the final model into an easy‐to‐use prediction model (Figure S2), but the model is not limited to maternal surveillance, as it also addresses PE‐associated fetal risk requiring delivery.

There has been a significant amount of research on the ability of the sFlt‐1/PlGF ratio to predict the absence or presence of PE or PE‐related complications, and different cut‐off values have been proposed^10^, ^11^, ^12^, ^13^, ^14^. We reported previously that the prediction of PE‐related complications could be improved significantly by using continuous instead of dichotomous values of biomarkers^7^. Based on this finding, the sFlt‐1/PlGF ratio is used as a continuous variable in our prediction model, which may provide an aid for obstetricians to better predict which patients are at high risk of serious maternal and fetal PE‐related complications, i.e. those who should be admitted to the obstetric ward, and which patients are at a low risk and can be followed at the outpatient clinic. This may reduce not only unnecessary preterm deliveries, antenatal admissions and fetal monitoring with false‐positive diagnoses, but possibly also costs by limiting the number of procedures performed because of diagnostic uncertainty. Of course, this should be balanced against the additional costs related to the measurement of the biomarkers.

For better assessment of its value, evaluation of our model in an external cohort would be worthwhile, but we have not yet been successful in doing so. If comparable c‐statistics were achieved on external validation, we could conclude with more certainty that our model is generalizable to other populations of women with suspected or confirmed PE. Importantly, the patients on whom our clinical prediction model is based were from one academic hospital and two general hospitals, providing a mix of more‐ and less‐severe cases.

A crucial question is how our model can be used in clinical practice. After discussion with a group of obstetricians in our hospital, we concluded that patients with suspected or confirmed PE who have a calculated risk of < 5% (sensitivity of 98% and thus a low false‐negative rate) are at low risk of maternal and fetal complications, and can be followed safely at the outpatient clinic. We acknowledge that this threshold of < 5% has been chosen arbitrarily. To establish whether this threshold is valid and safe for use in clinical practice, we commenced a randomized intervention trial. In this trial, patients with suspected or confirmed PE are randomized to management according either to existing guidelines or to treatment based on the clinical prediction model. From this, it will be possible to determine whether introduction of the prediction model is safe and a welcome aid for obstetricians in providing care for patients with (suspected) PE and whether it would result in fewer hospital admissions and additional investigations and measurements, with the potential for saving costs. As for its ease of use in a clinical setting, the prediction model in Excel is generally perceived to be convenient by the participating hospitals.

### Strengths and limitations

A strength of this study is that we, for the first time, have developed a model to predict a composite outcome of PE‐related pregnancy complications that consists of maternal and fetal adverse outcomes, within 7, 14 and 30 days, in women with suspected or confirmed PE, rather than a model only for diagnosing PE. The current study also expands on former studies by the ease of use of the prediction model in a clinical setting based on commercially available and fully automated immunoassays.

There are several limitations of this study, including the lack of external validation. External validation may ensure that predictions based on the developed clinical prediction model are generalizable to other populations. Second, our model is based on a relatively small sample size, which might have resulted in some degree of overfitting, despite our internal validation with bootstrap resampling. Furthermore, to be useful in clinical decision‐making for patients with suspected or confirmed PE presenting on the obstetric ward, it is important that the biomarkers can be measured instantaneously as a point‐of‐care measurement, which requires a dedicated clinical chemistry laboratory.

### Perspectives

We present a multivariable prediction model with an additional tool to calculate the absolute risk of developing a composite outcome of PE‐related pregnancy complications, consisting of maternal and fetal adverse events, in the subsequent 7, 14 and 30 days, in women with suspected or confirmed PE. This information could help physicians to identify low‐risk patients who could be offered expectant management, leading to a decrease in the number and duration of admissions to the obstetric ward and potentially a reduction in costs, while simultaneously not compromising maternal and fetal health outcomes. Furthermore, we expect that implementation of the prediction model will improve the timing of delivery, resulting in improved maternal and neonatal health.

## Supporting information


**Figure S1** Flowchart of the study design. *Blood was drawn at study entry, but soluble fms‐like tyrosine kinase‐1 (sFlt‐1) and placental growth factor (PlGF) were measured after delivery to prevent any influence of this information on decision‐making. GA, gestational age at study entry; PE, pre‐eclampsia.

**Figure S2** Details of the algorithm of the prediction model. (a) By entering in the Excel calculator the gestational age in weeks and days (which will automatically be converted to days in the algorithm), the protein‐to‐creatinine ratio (in mg/mmol) and the soluble fms‐like tyrosine kinase‐1/placental growth factor (sFlt‐1/PlGF) ratio, a calculated percentage risk will be displayed for the development of pre‐eclampsia (PE)‐related maternal/fetal complications within 7, 14 and 30 days. (b–d) Example risk results for low protein‐to‐creatinine ratio of 10 and low sFlt‐1/PlGF ratio of 10 (b), elevated protein‐to‐creatinine ratio of 38 and low sFlt‐1/PlGF ratio of 10 (c) and low protein‐to‐creatinine ratio of 10 and elevated sFlt‐1/PlGF ratio of 38 (d).

## Data Availability

The data that support the findings of this study are available from the corresponding author upon reasonable request. The data are not publicly available due to privacy or ethical restrictions.
