# Supplementary material for: Prediction of pre‐eclampsia‐related complications in women with suspected or confirmed pre‐eclampsia: development and internal validation of clinical prediction model
Source: Ultrasound Obstet Gynecol. 2021 Oct 6;58(5):698–704. doi: 10.1002/uog.23142 (PMC8596877; doi:10.1002/uog.23142)
Supplement: Supplementary file 1 — Figure S1 Flowchart of the study design. *Blood was drawn at study entry, but soluble fms‐like tyrosine kinase‐1 (sFlt‐1) and placental growth factor (PlGF) were measured after delivery to prevent any influence of this information on decision‐making. GA, gestational age at study entry; PE, pre‐eclampsia. [file UOG-58-698-s002.pptx]

## Slide 1
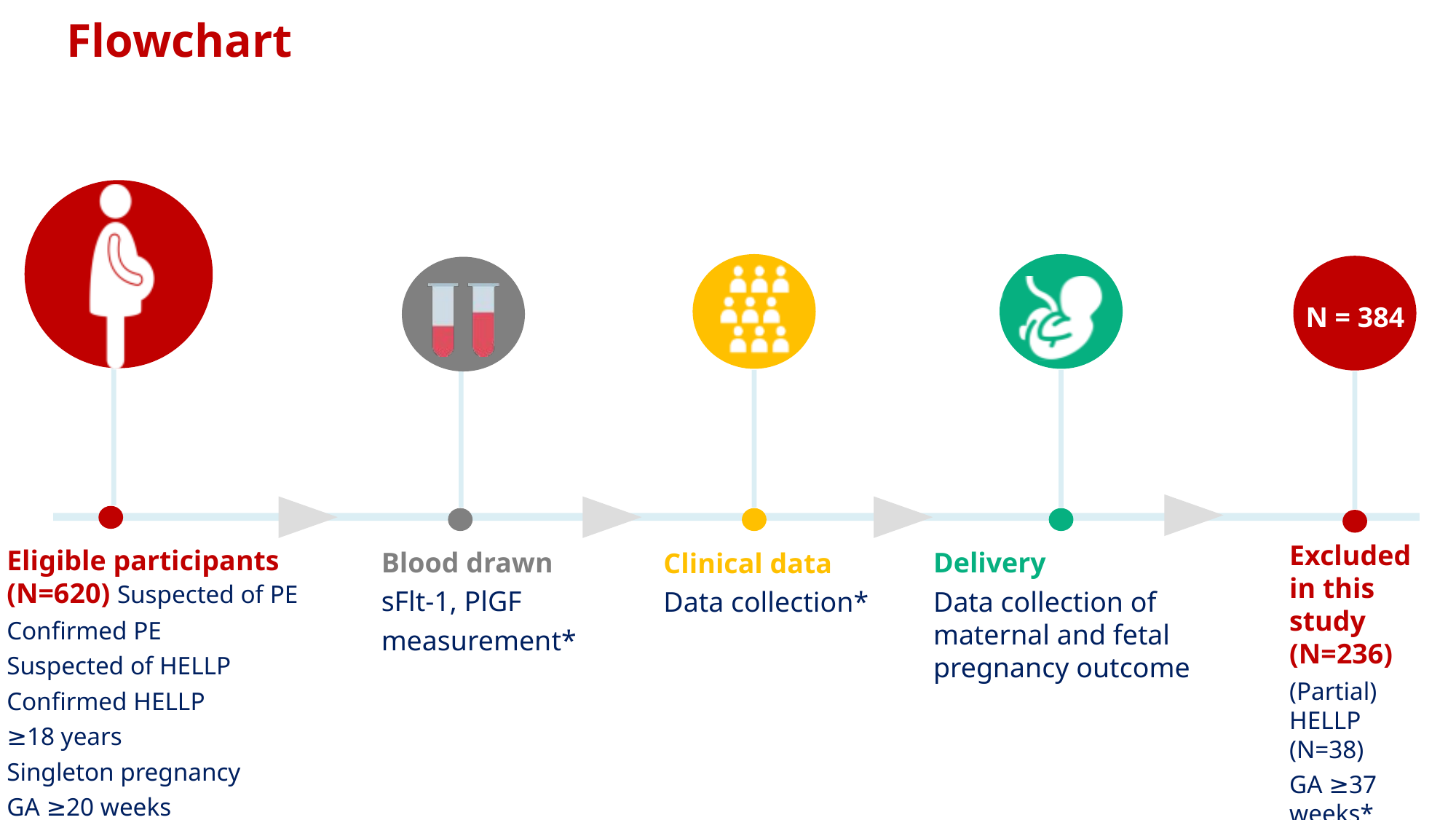

# Flowchart
Eligible participants (N=620) Suspected of PE
Confirmed PE
Suspected of HELLP
Confirmed HELLP
≥18 years
Singleton pregnancy
GA ≥20 weeks
N = 384
Excluded in this study (N=236)
(Partial) HELLP (N=38)
GA ≥37 weeks* (N=198)
Delivery
Data collection of maternal and fetal pregnancy outcome
Blood drawn
sFlt-1, PlGF
measurement*
Clinical data
Data collection*
