# Supplementary material for: Prediction of pre‐eclampsia‐related complications in women with suspected or confirmed pre‐eclampsia: development and internal validation of clinical prediction model
Source: Ultrasound Obstet Gynecol. 2021 Oct 6;58(5):698–704. doi: 10.1002/uog.23142 (PMC8596877; doi:10.1002/uog.23142)
Supplement: Supplementary file 2 — Figure S2 Details of the algorithm of the prediction model. (a) By entering in the Excel calculator the gestational age in weeks and days (which will automatically be converted to days in the algorithm), the protein‐to‐creatinine ratio (in mg/mmol) and the soluble fms‐like tyrosine kinase‐1/placental growth factor (sFlt‐1/PlGF) ratio, a calculated percentage risk will be displayed for the development of pre‐eclampsia (PE)‐related maternal/fetal complications within 7, 14 and 30 days. (b–d) Example risk results for low protein‐to‐creatinine ratio of 10 and low sFlt‐1/PlGF ratio of 10 (b), elevated protein‐to‐creatinine ratio of 38 and low sFlt‐1/PlGF ratio of 10 (c) and low protein‐to‐creatinine ratio of 10 and elevated sFlt‐1/PlGF ratio of 38 (d). [file UOG-58-698-s001.doc]

**Figure S2** Details of the algorithm of the prediction model.


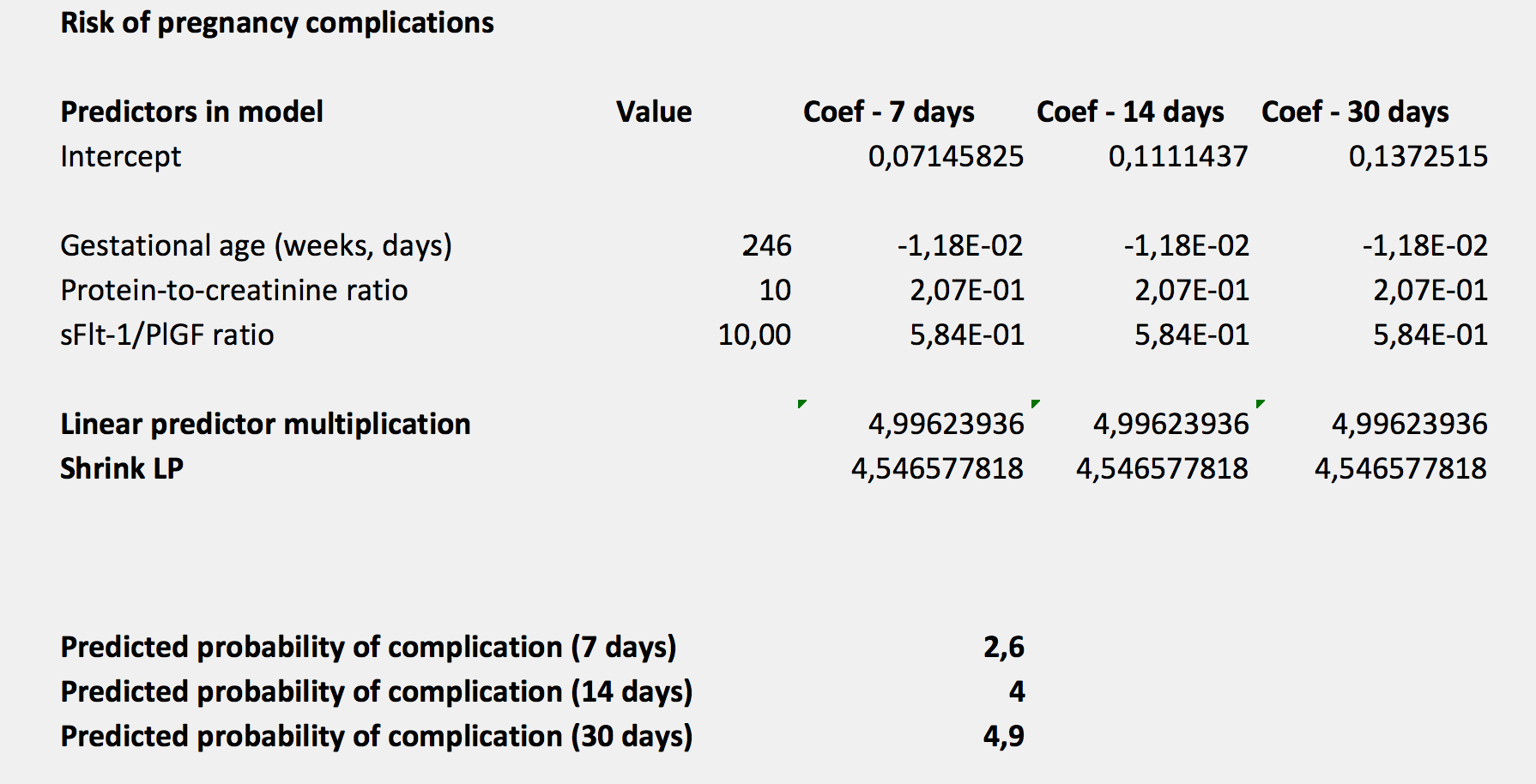

(a) By entering the gestational age (in weeks and days), the protein-to-creatinine ratio (mg/mmol), and the sFlt-1/PlGF ratio, a calculated risk as a percentage will be displayed for 7, 14 and 30 days. LP indicates linear prediction.


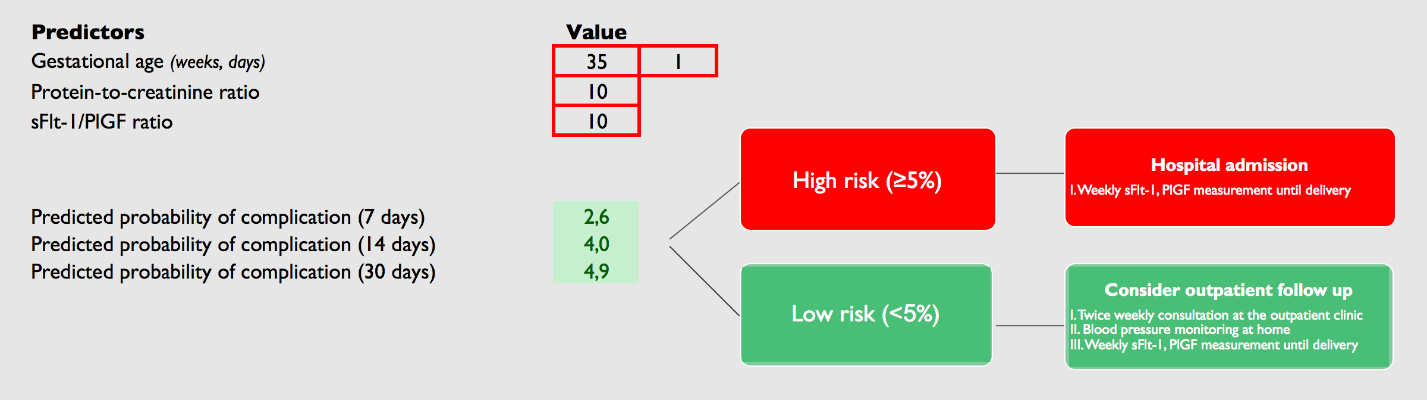
(b) low protein-to-creatinine ratio of 10 and low sFlt-1/PlGF ratio of 10.


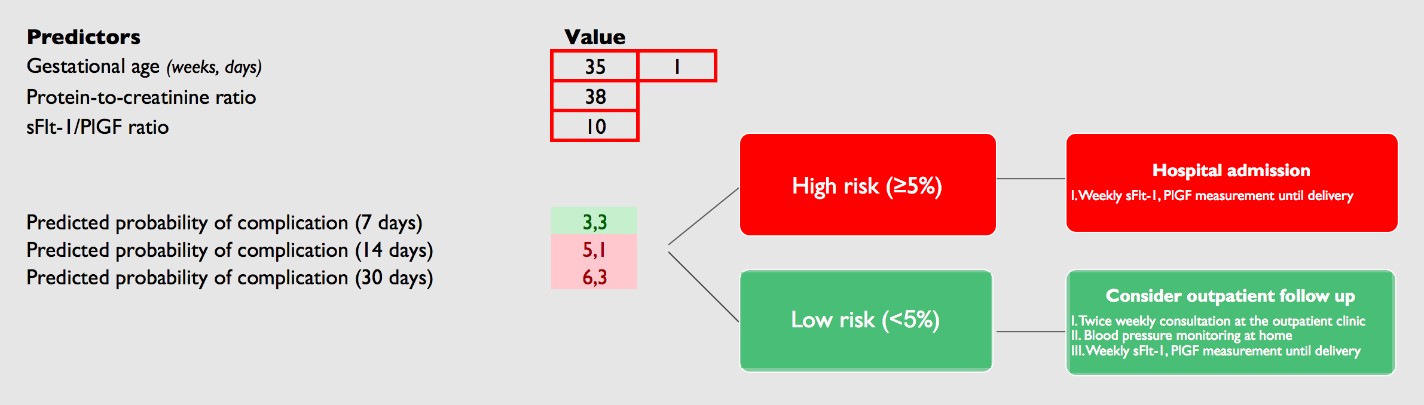

(c) elevated protein-to-creatinine ratio of 38 and low sFlt-1/PlGF ratio of 10.


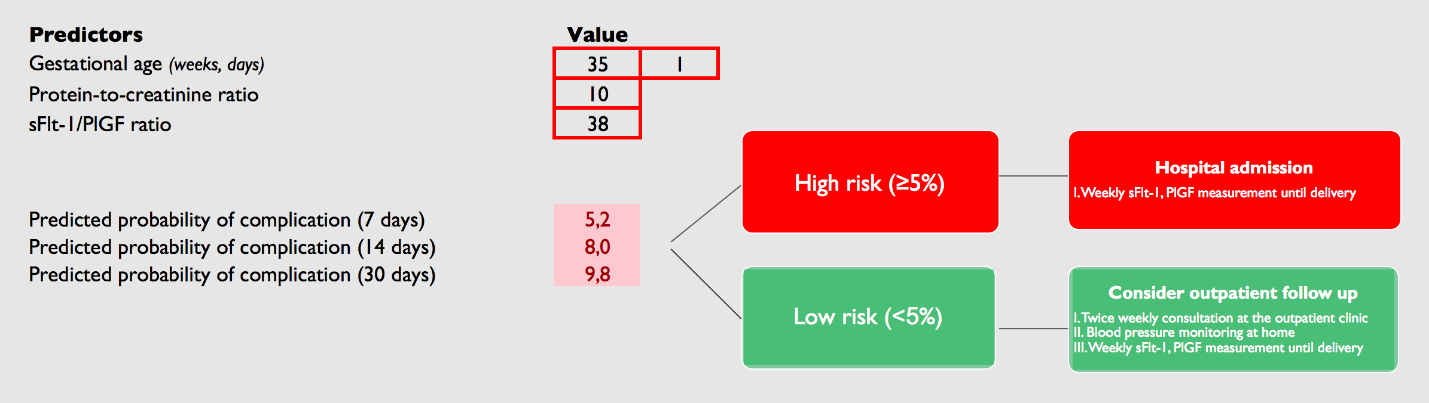


(d) low protein-to-creatinine ratio of 10 and elevated sFlt-1/PlGF ratio of 38.
